# The Eastern Pest

**Published:** 1898-11

**Authors:** 


THE EASTERN PEST.—"BUBONIC PLAGUE."—The death of an eminent scientist in Vienna recently from accidental inoculation with the disease while experimenting with a "culture," has brought the subject again prominently to the front, and fears are entertained that in view of the complications now existing amongst certain nations, and which may lead to war, the infection will become widely disseminated. If so, it will have a demoralizing effect, for the bravest soldier, one who will face fire and brimstone, shot, shell and *official incompetence,* will blanch before and shrink from the man on the white horse when he rides on such a mount. The *doctors* will face it, however,—never fear; and sooner or later, science will overthrow this rough rider,—tho' many brave ones will fall first.

---

THE DEATH OF COL. WARING in New York October 30, from yellow fever contracted in Havana, is a most lamentable occurrence. His loss is irreparable. He would, had he lived, have instituted measures in Cuba which would have eradicated the yellow fever. As a sanitary engineer he had not an equal; he was a sanitary Edison or Tesla,—a Napoleon of sanitary engineering. I do not know any one who can fill his place. The "Waring system of sewers" became world famous after its introduction at Memphis, Tenn., in 1880. Memphis had been the deadliest place known. After Waring's great work there the death rate from zymotic disease was reduced fifty per cent.

